# Energetics Equivalent of the Cardiac Force-Length End-Systolic Zone: Implications for Contractility and Economy of Contraction

**DOI:** 10.3389/fphys.2019.01633

**Published:** 2020-01-21

**Authors:** Kenneth Tran, Andrew J. Taberner, Denis S. Loiselle, June-Chiew Han

**Affiliations:** ^1^Auckland Bioengineering Institute, The University of Auckland, Auckland, New Zealand; ^2^Department of Engineering Science, The University of Auckland, Auckland, New Zealand; ^3^Department of Physiology, The University of Auckland, Auckland, New Zealand

**Keywords:** cardiac energetics, shortening heat, cardiac mechanics, force-length relation, cardiac end-systolic zone

## Abstract

We have recently demonstrated the existence of a region on the cardiac mechanics stress-length plane, which we have designated “The cardiac end-systolic zone.” The zone is defined as the area on the pressure-volume (or stress-length) plane within which all stress-length contraction profiles reach their end-systolic points. It is enclosed by three boundaries: the isometric end-systolic relation, the work-loop (shortening) end-systolic relation, and the zero-active stress isotonic end-systolic relation. The existence of this zone reflects the contraction-mode dependence of the cardiac end-systolic force-length relations, and has been confirmed in a range of cardiac preparations at the whole-heart, tissue and myocyte levels. This finding has led us to speculate that a comparable zone prevails for cardiac metabolism. Specifically, we hypothesize the existence of an equivalent zone on the energetics plane (heat vs. stress), and that it can be attributed to the recently-revealed heat of shortening in cardiac muscle. To test these hypotheses, we subjected trabeculae to both isometric contractions and work-loop contractions over wide ranges of preloads and afterloads. We found that the heat-stress relations for work-loop contractions were distinct from those of isometric contractions, mirroring the contraction mode-dependence of the stress-length relation. The zone bounded by these contraction-mode dependent heat-stress relations reflects the heat of shortening. Isoproterenol-induced enhancement of contractility led to proportional increases in the zones on both the mechanics and energetics planes, thereby supporting our hypothesis.

## Introduction

The force produced by an isometrically contracting muscle can be varied over a range of initial lengths or preloads. Contractility is illustrated on the force-length plane as the slope of the isometric end-systolic force-length relation. As first revealed by Frank in his cardiac pressure-volume diagram (Frank, 1899), a separate relation in the plane exists for a series of afterloaded shortening (work-loop) contractions. These two force-length relations (isometric and work-loop) are distinct – that for isometric contractions lying above its work-loop counterpart. The divergence of these two force-length relations, exemplified particularly under conditions of either high preloads or low afterloads, reveals a region that encompasses all possible end-systolic force-length points. This zone has been coined the “cardiac end-systolic zone,” and is applicable to preparations ranging from the whole-heart (Frank, 1899; Kissling et al., 1985) to isolated cardiac tissues (Gülch and Jacob, 1975; Han et al., 2019) and myocytes (Iribe et al., 2014), as illustrated in Figure 7 of Han et al. (2019). For over a century, it was unclear why some experimentalists observed a single end-systolic relation for both isometric and afterloaded shortening contractions while others observed two separate relations. The demonstration of the existence of a cardiac end-systolic zone by Han et al. (2019) has resolved this long-standing puzzle in the field of cardiac mechanics.

The mechanical properties of cardiac muscle described above are intimately linked with its energetics characteristics. We therefore hypothesize that there exists an equivalent zone for energetics, specifically on the heat-force plane. An isometrically-contracting muscle transduces chemical energy entirely into heat, whereas an afterloaded shortening muscle captures some of that energy as work (Gibbs, 1978). When assessed on the heat-force plane, the mechanoenergetics of cardiac muscle likewise displays contraction-mode dependence (Pham et al., 2017; Tran et al., 2017). Explicitly, for a given force, the heat arising from an isometric contraction is less than that from a work-loop contraction, resulting in the isometric heat-force relation lying below its work-loop counterpart. We further hypothesize that the region lying between these two contraction mode-dependent heat-force relations reflects the existence of “heat of shortening” in cardiac muscle (Tran et al., 2017). That is, the energetics equivalent of the end-systolic zone (ESZ) on the mechanics plane is the “heat of shortening” region, and this region is also expected to encompass all possible heat-force points resulting from isometric and work-loop contractions. We tested this proposed equivalence by simultaneously measuring force, length, and heat under both isometric and work-loop modes of contraction and over a wide range of preloads and afterloads. We further challenged their equivalence by the use of an inotropic agent that increases cardiac contractility.

## Materials and Methods

This study was carried out in accordance with the principles of the Basel Declaration. The protocol was approved by the University of Auckland Animal Ethics Committee under the Approval Number R2006. Following anesthesia with isoflurane (5% in O_2_), each rat (8–10 week-old male Wistar strain of weights ranging from 300 to 350 g) was injected with heparin (1000 IU kg^–1^) and killed via cervical dislocation. The heart was excised and immediately arrested by submergence in chilled Tyrode solution. The aorta was rapidly cannulated and Langendorff-perfused with low-Ca^2+^ Tyrode solution (in mmol l^–1^: 130 NaCl, 6 KCl, 1 MgCl_2_, 0.5 NaH_2_PO_4_, 0.3 CaCl_2_, 10 Hepes, 10 glucose, 20 2,3-butanedione monoxime, pH adjusted to 7.4 using Tris), and vigorously gassed with 100% O_2_ at room temperature.

Experiments were performed on *n* = 12 trabeculae dissected from the left ventricle. Six trabeculae from four rats were randomly assigned to a Control group, where they were exposed to Tyrode solution with composition as listed above except for a higher concentration of CaCl_2_ (1.5 mmol l^–1^) and in the absence of 2,3-butanedione monoxime. The other six trabeculae from the other four rats were assigned to an Isoproterenol group, where they were exposed to the same Tyrode solution with addition of the inotropic agent isoproterenol (0.5 μmol l^–1^). The isoproterenol dose-response curve for active force development saturates at concentrations above 0.1 μmol l^–1^ (Monasky et al., 2008). We chose a concentration of 0.5 μmol l^–1^ to ensure that the maximum inotropic response is elicited, which is within the typical range, 0.1 μmol l^–1^ (Barclay et al., 1979) to 1 μmol l^–1^ (Power et al., 2018), used for cardiac muscle experiments.

Each trabecula was mounted in our flow-through work-loop calorimeter (Taberner et al., 2011, 2018) and superfused with Tyrode solution. After a period of about 1 h of continuous 2 Hz electrical stimulation, during which mechanical performance of the trabecula and thermal equilibration of the apparatus were reached, the muscle was slowly lengthened to optimal length (*L*_o_) where twitch force was maximal. Muscle dimensions (*L*_o_, and the top and perpendicular views of diameter) were measured with a microscope graticule. There were no statistically significant differences in trabecula dimensions between the Control group and the Isoproterenol group in either cross-sectional area (0.08 ± 0.01 and 0.06 ± 0.01 mm^2^, respectively) or length (3.2 ± 0.2 and 2.8 ± 0.2 mm, respectively).

The entire calorimeter apparatus was enclosed within a hood to diminish external optical and thermal disturbances. Ambient temperature within the hood was maintained at 32°C by temperature controllers installed on the top and at the bottom of the measurement chamber, and a separate system heating the optical table upon which the entire calorimeter system is mounted.

Experiments commenced when a stable thermal environment was reached. Stimulus frequency was then increased to 3 Hz. The trabecula was first subjected to a series of five shortening contractions at progressively diminishing afterloads, thereby performing a series of afterloaded work-loops (see Taberner et al., 2011 for details). This series of five afterloaded work-loop contractions was further performed at two reduced muscle lengths (0.95 *L*_o_ and 0.90 *L*_o_). At all three lengths (preloads), isometric contractions were interspersed between each of the five afterloaded work-loop contractions. Following the three preloaded, five afterloaded, work-loop contractions, muscle length was further reduced to 0.85 *L*_o_, 0.81 *L*_o_, and 0.77 *L*_o_ to perform additional isometric contractions. Completion of the experimental protocol thus included isometric twitches obtained at six muscle lengths.

The rate of heat production (measured in units of W) was recorded simultaneously alongside twitch force and muscle length, and subsequently converted to heat per twitch (expressed in units of J) by dividing by the stimulus frequency. Upon completion of an experiment, two sources of heat artifact were quantified while the trabecula was unstimulated, thereby remaining in its quiescent state. The heat artifact arising from the cyclic movement of the upstream hook (required for muscle shortening during work-loop contractions) was quantified with the muscle quiescent by oscillating the hook at a frequency of 3 Hz over the extent of muscle shortening achieved at the lowest afterload. The heat artifact resulting from electrical stimulation was quantified by electronically relocating the muscle downstream, away from the measurement chamber and into the mounting chamber. Both heat artifacts were subtracted from the measured heat signal before further data analyses.

### Statistical Analyses

Data were fitted using polynomial regression (up to third order) and the regression lines were averaged within groups using the “random coefficient model” within PROC MIXED of the SAS software package. The significance of differences among regression lines, or between mean values, of the two groups was tested and declared when *P* < 0.05.

## Results

**FIGURE 1:**
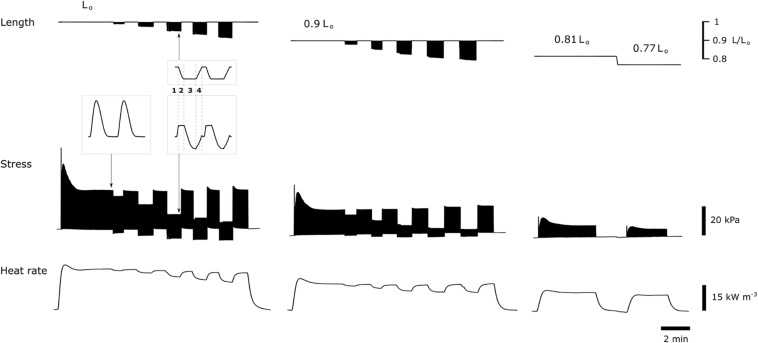
Subset of experimental records. Muscle length, muscle stress, and associated heat signal were simultaneously recorded. The **left panel** shows records of combined isometric and work-loop contractions, with the former mode interposed with latter, from an initial length at *L*_o_. Work-loop contractions with the five decreasing afterloads are concomitant with increasing extent of shortening and decreasing output of heat. The **middle panel** shows records at an initial length of 0.9 *L*_o_. The **right panel** shows records of two isometric contractions at 0.81 *L*_o_ and 0.77 *L*_o_. Electrical stimulation was paused between interventions required for the reduction of initial length. Insets show expanded traces for two individual twitches at steady state, indicated by the arrows. The work-loop twitch consists of four phases: (1) isometric contraction, (2) isotonic shortening, (3) isometric relaxation, and (4) passive re-stretch back to initial muscle length.

Figure 1 shows a subset of experimental records of simultaneous measurements of muscle length, muscle stress and heat signal. At each initial length (preload), isometric stress and heat were the highest compared with those achieved in the work-loop contractions where reducing afterload resulted in decreasing heat but increasing extent of shortening. With decreasing initial length (preload), both isometric stress and heat reduced.

**FIGURE 2:**
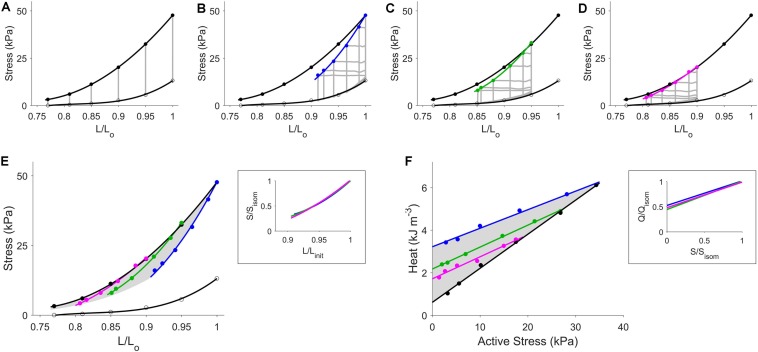
Steady-state data from a representative trabecula superfused with Tyrode solution. In panel **(A)**, the isometric end-systolic stress-length relation (upper thick line) and the isometric passive stress-length relation (lower thick line) have been fitted to the six isometric contractions (gray lines) at end-systole (black circles) and at end-diastole (open circles), respectively. These two relations have been transcribed to panels **(B–E)**. The work-loop end-systolic stress-length relation, obtained by fitting to the end-systolic points (filled circles), is dependent on the initial length (lines: blue, green, and magenta, respectively): at *L*_o_ **(B)**, 0.95 *L*_o_ **(C)**, and 0.90 *L*_o_ **(D)**. In panel **(E)**, all stress-length relations are superimposed, where the shaded area indicates the zone of the end-systolic points as interpolated using the self-normalized work-loop stress-length relations in the inset, in which *L*_init_ refers to the three initial muscle lengths (*L*_o_, 0.95 *L*_o_, and 0.90 *L*_o_). In panel **(F)**, the shaded area indicates the zone of the heat-stress relations obtained from the three preloaded work-loop contractions and the isometric contractions. The inset shows the three relations between work-loop heat (Q) and stress (S) normalized to their isometric values (subscripted as “isom”).

Figure 2 shows results from a single, representative, trabecula in the Control group. Panel A shows the isometric end-systolic (upper thick line) and end-diastolic (passive; lower thick line) stress-length relations. Both relations were obtained by fitting to their respective six end-systolic points (black circles) and end-diastolic points (open circles) of the isometric contractions at steady state (vertical gray lines). These isometric stress-length relations are replicated in panels B–E of Figure 2. Panels B–D show, respectively, the work-loop contractions and their corresponding end-systolic stress-length relations (ESSLRs) at three initial muscle lengths (preloads): *L*_o_, 0.95 *L*_o_, and 0.90 *L*_o_ (blue, green, and magenta curves). Note that these work-loop ESSLRs each terminated at its own unique lowest achievable afterload. These lowest afterloads fell in the vicinity of their respective passive stresses. These results reveal that the ESSLR is indeed *dependent* on the mode of contraction as the three work-loop ESSLRs each diverged from the isometric ESSLR. The magnitude of their divergence from the isometric ESSLR decreased with decreasing preload, as is evident in Figure 2E where the three work-loop ESSLRs (colored) are superimposed, along with the isometric stress-length relations (black). The curvatures of each of the three work-loop ESSLRs were not significantly different from one another, as more clearly revealed in the inset of panel E where they have been self-normalized to both the isometric stress and the initial length. Using the curvature, we interpolated the work-loop ESSLR for any given initial muscle length, resulting in the shaded area which we have coined the “cardiac end-systolic zone” in the stress-length plane.

**FIGURE 3:**
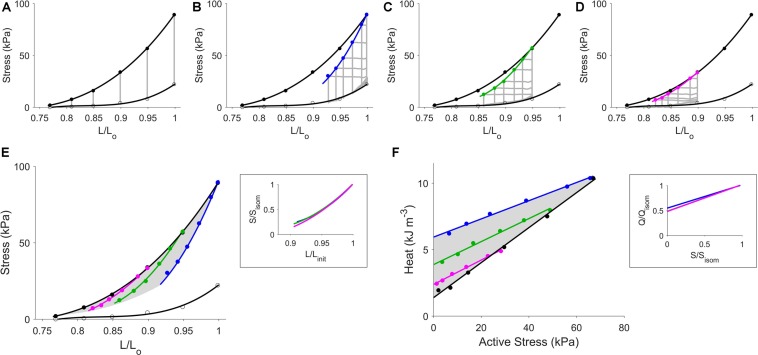
Steady-state data from a representative trabecula superfused with Tyrode solution and isoproterenol. Figure caption is the same as that in Figure 2. In panel **(A)**, the isometric end-systolic stress-length relation (upper thick line) and the isometric passive stress-length relation (lower thick line) have been fitted to the six isometric contractions (gray lines) at end-systole (black circles) and at end-diastole (open circles), respectively. These two relations have been transcribed to panels **(B–E)**. The work-loop end-systolic stress-length relation, obtained by fitting to the end-systolic points (filled circles), is dependent on the initial length (lines: blue, green, and magenta, respectively): at *L*_o_ **(B)**, 0.95 *L*_o_ **(C)**, and 0.90 *L*_o_ **(D)**. In panel **(E)**, all stress-length relations are superimposed, where the shaded area indicates the zone of the end-systolic points as interpolated using the self-normalized work-loop stress-length relations in the inset, in which *L*_init_ refers to the three initial muscle lengths (*L*_o_, 0.95 *L*_o_, and 0.90 *L*_o_). In panel **(F)**, the shaded area indicates the zone of the heat-stress relations obtained from the three preloaded work-loop contractions and the isometric contractions. The inset shows the three relations between work-loop heat (Q) and stress (S) normalized to their isometric values (subscripted as “isom”).

In panel F of Figure 2, a zone equivalent to the “cardiac end-systolic zone” in the stress-length plane (panel E) was obtained. This heat-stress zone (HSZ) shaded in gray on the heat-stress plane, likewise, contains the work-loop heat-stress relations where, again, their slopes were not significantly different from one another (as illustrated in the inset of panel F). Qualitatively similar results were obtained in a trabecula subjected to isoproterenol (Figure 3).

**FIGURE 4:**
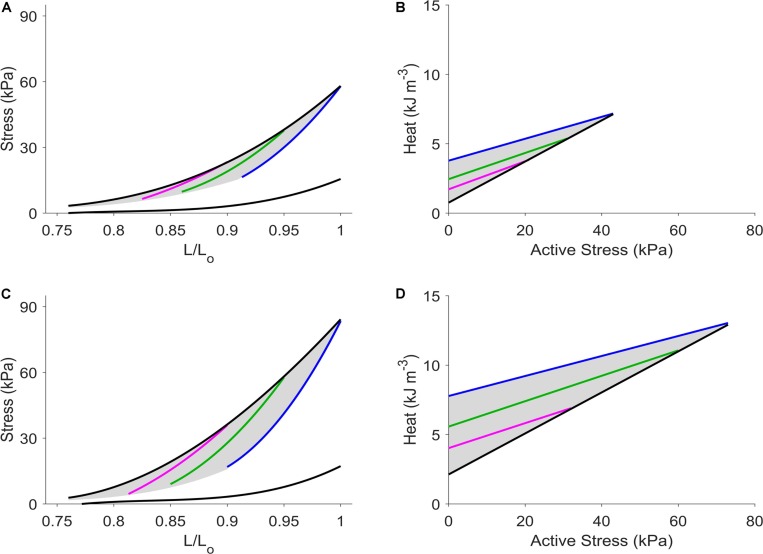
Average regression lines from six trabeculae per group. Average lines from the Control group are presented in panels **(A,B)**, and those from the Isoproterenol group are depicted in panels **(C,D)**. Black lines denote isometric contraction. Blue, green, and magenta, respectively, denote work-loop contractions at *L*_o_, 0.95 *L*_o_, and 0.90 *L*_o_. The shaded areas in **A** and **C** denote the end-systolic zones, whereas those in **B** and **D** denote the heat-stress zones.

Figure 4 shows the average results from six trabeculae in the Control group (panels A and B) and from a different set of six trabeculae in the Isoproterenol group (panels C and D). The equivalence between the ESZ in the stress-length plane and the zone in the heat-stress plane was preserved under the isoproterenol challenge.

**FIGURE 5:**
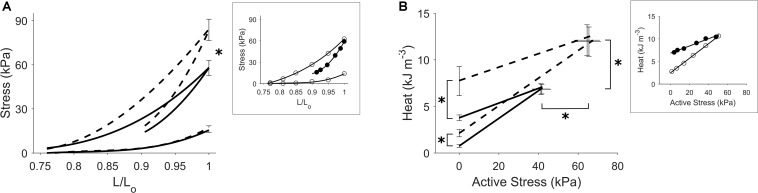
Effects of increased contractility. Average regression lines from trabeculae in the Control group (continuous lines) and those in the Isoproterenol group (broken lines) undergoing preloaded isometric contractions and afterloaded work-loop contractions at *L*_o_ are shown in each panel. The asterisk in panel **(A)** indicates a statistically significant difference between the regression lines. The asterisks in panel **(B)** signify effects of isoproterenol on the heat-intercepts as well as on the peak active stress and muscle heat output. Standard errors of means at peak values are superimposed in all panels. The insets depict regression lines fitted to the data from a representative Control trabecula where open circles denote isometric contractions and filled circles denote work-loop contractions at *L*_o_.

**FIGURE 6:**
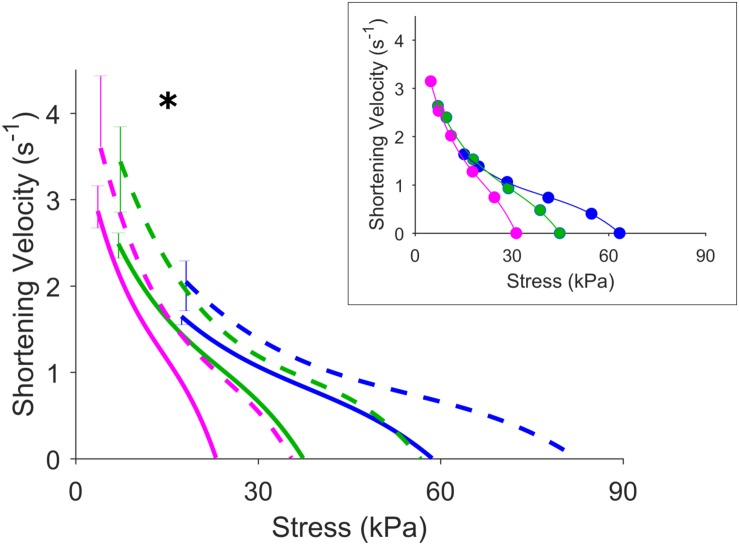
Effects of increased contractility on velocity of shortening. Inset shows representative data for a Control muscle. The asterisk indicates that the velocity of shortening at the lowest afterload of work loop contraction for the three initial muscle lengths is greater for the Isoproterenol group (dashed lines) compared with the Control group (solid lines). Blue, green, and magenta colored lines represent work-loop contractions starting at *L*_o_, 0.95 *L*_o_, and 0.90 *L*_o_, respectively.

Figure 5 shows the effects of isoproterenol on mechanoenergetics under both preloaded isometric contractions and afterloaded work-loop contractions at *L*_o_. Trabeculae exposed to isoproterenol had elevated isometric ESSLR and work-loop ESSLR, with no difference in their passive stress-length relations (panel A). In panel B, both the isometric heat-stress relation and the work-loop heat-stress relation were significantly higher in the Isoproterenol group, as evident by the higher heat-intercepts and higher isometric active stress and isometric heat. The Isoproterenol group had greater shortening kinetics, producing higher velocities of shortening across all three initial muscle lengths (Figure 6).

## Discussion

In this study, we extended the ESZ framework developed for cardiac mechanics to encompass energetics. The ESZ framework revealed the existence of a zone on the force-length plane within which all end-systolic points are situated. The zone is bounded above by the isometric ESSLR, and below by work-loop contractions at *L*_o_ and work-loops resulting from zero-active force. Within these three boundaries exists an infinite number of work-loop ESFLRs, each unique to a given preload. We show in Figure 2F that these features, observed in the mechanics plane, are mirrored on the energetics plane in the form of an equivalent HSZ. As is the case with mechanics, the heat-stress relations are dependent on the mode of contraction. The isometric heat-stress relation (black line) sits below all those arising from work-loop contractions (colored lines). For work-loop contractions at reduced preloads, their heat-stress relations are located progressively closer to the isometric heat-stress relation. The entire HSZ is bounded above by work-loop contractions at *L*_o_, and below by isometric contractions at different muscle lengths. The HSZ encompasses all possible heat-stress points for a given level of contractility, consistent with the property of the ESZ of encompassing all end-systolic points. When muscle contractility was increased via addition of isoproterenol, new commensurate zones for ESZ and HSZ were established, demonstrating an inotropic dependence inherent in both zones, thereby further strengthening our claim of their equivalence (Figure 4).

### Shortening Heat

In skeletal muscle, the hydrolysis of ATP associated with muscle shortening leads to the production of work and the liberation of heat above that of isometric contractions. This “extra” heat above the isometric heat was termed the “heat of shortening” by Hill (1938). Until recently, it was thought that the heat of shortening is a property unique to skeletal muscle (Gibbs and Chapman, 1979; Holroyd and Gibbs, 1992). However, a recent experimental and modeling study (Tran et al., 2017) has provided compelling evidence that the heat of shortening also exists in cardiac tissues. At any given force, more heat is produced from a shortening contraction than from an isometric contraction owing to the liberation of heat from muscle shortening. In this study, we find that the HSZ describes the liberation of heat which is in excess of heat produced by isometric contractions, consistent with the heat of shortening. Given that the ESZ arises from the difference between the end-systolic points of isometric and shortening contractions on the mechanics plane, it is not surprising that its energetic equivalent is the difference in heat liberated by these two modes of contraction. The ESZ and HSZ represent two orthogonal quantifications of particular characteristics in a cardiac muscle twitch. That is, a point within the ESZ marks the moment of end-systole of a twitch on the stress-length plane, while a point within the HSZ quantifies the heat liberated over the course of the twitch on the heat-stress plane.

### Implications for Economy of Contraction

The inverse of the slope of the heat-stress relation is a measure of the economy of contraction (Gibbs, 1978). It is typically quantified from isometric contractions in order to relate muscle force production to its energetic cost (Gibbs, 1978; Alpert and Mulieri, 1982; Han et al., 2010; Johnston et al., 2016). Figure 4B shows that the slope of the heat-stress relation for isometric contractions is steeper than its work-loop counterpart, giving the impression of a higher economy of contraction under work-loop contractions. But this impression is misleading because the work-loop heat-stress relation contains heat that is in excess of isometric cross-bridge heat (*Q*_XB(Isom)_), in accord with Figure 10 of Tran et al. (2017). The partitioning of cross-bridge heat in is this manner demonstrates that more heat is liberated for a shortening contraction than an isometric contraction at a given level of force (Rall, 1982). However, this is not to say that in a shortening muscle, there are a subset of cross-bridges that cycle isometrically while another subset is involved in sarcomere shortening. *Q*_XB(Isom)_ can be measured only from muscles undergoing isometric contractions. In our opinion, economy of contraction should be strictly derived from *Q*_XB(Isom)_ and therefore be purely a property of isometric contractions.

We also observed that the economy of contraction is independent of an increase in contractility induced by the addition of the beta-adrenergic stimulant, isoproterenol. The slopes of the isometric heat-stress relations for the control and isoproterenol cases were the same despite an increase in force in the isoproterenol case (Figure 5B). This suggests that isoproterenol has no direct effect on the chemo-mechanical energy transduction of cardiac myofilaments and that the augmentation of contractility arises indirectly from an increase in the peak of the cytosolic Ca^2+^ transient (Tamura et al., 1992; Kassiri et al., 2000; Power et al., 2018). Our measurement of a null effect of isoproterenol on economy, but a higher activation heat in the presence of isoproterenol, is consistent with earlier observations reported in the literature (Gibbs, 1967; Gibbs and Gibson, 1972; Barclay et al., 1979).

The preservation of the economy of contraction in the presence of an isoproterenol-induced increase in contractility suggests that the efficiency of the myofibrillar energy transduction step has not been altered (Gibbs and Gibson, 1972). That is, the efficiency of ATP hydrolysis by myosin ATPase is unaffected by isoproterenol despite an increase in the velocity of shortening (Figure 6). The faster shortening kinetics is consistent with reported effects of the beta-adrenergic stimulant increasing cross-bridge cycling rates in both intact (Hoh et al., 1988; Kentish et al., 2001) and skinned cardiac preparations (Strang et al., 1994; Saeki et al., 1997). On the surface, these results appears to be at odds with reports that slowing of cross-bridge kinetics is a compensatory mechanism that increases economy of contraction in hypertrophic (Alpert and Mulieri, 1982), diabetic (Rundell et al., 2004), and aging (van der Velden et al., 1998; Kiriazis and Gibbs, 2001) hearts. Slowing of cross-bridge cycling kinetics is typically associated with a shift of myosin isoforms. The two types of myosin heavy chains (MHC) in the heart (α-MHC and β-MHC) carry the site for ATPase activity (Hoh et al., 1978). Their formation of homo- or heterodimers give rise to, in order of decreasing ATPase activity, V1, V2, and V3 myosin isoforms (Pope et al., 1980). In aging and diseased hearts, remodeling of the tissue leads to replacement of the myosin V1 isoform with the kinetically slower V3 isoform. Given the difference in ATPase activity between these myosin isoforms, we speculate that the myosin profile of the tissue is the key determinant of contraction economy (Rundell et al., 2004; Narolska et al., 2005a, b), rather than cycling kinetics *per se*.

### Implications for Contractility

The effect of isoproterenol to increase cardiac contractility has typically been demonstrated in intact isolated tissues contracting at a single fixed muscle length (Kassiri et al., 2000; Layland and Kentish, 2000; Power et al., 2018). Our isometric ESSLR protocol characterizes the peak stress development of twitching trabeculae over a range of muscle lengths in response to isoproterenol (Figure 5). An important implication of our ESZ framework is that an accurate assessment of changes in contractility requires identical loading conditions to be applied (Han et al., 2019). In Figure 5A, the change of contractility can be assessed by comparing either the isometric ESSLR or the work-loop ESSLR between the control and isoproterenol cases. Note that in each contraction mode the associated loading conditions were kept the same – i.e., isometric ESSLR up to *L*_o_ or work-loop ESSLR at an initial length of *L*_o_. Making these comparisons appropriately reveals steeper ESSLRs for muscles superfused with isoproterenol, indicative of an increase in contractility.

## Conclusion

In cardiac muscle, the ESZ is an area on the stress-length plane that encompasses all possible end-systolic points arising from isometric and work-loop contractions. The associated heat liberated from these two modes of contraction is circumscribed by a zone (HSZ) on an orthogonal heat-stress plane. These two zones are equivalent in that they are bounded by isometric contractions on one side and work-loop contractions at *L*_o_ on the other, and expand in the presence of an inotropic agent. The HSZ represents the heat of shortening associated with work-loop contractions. The equivalence between the mechanical and energetic properties of cardiac tissues has allowed us to comment on appropriate methods for comparing muscle contractility and assessing economy of contraction. We conclude that meaningful comparisons of contractility require parity in loading conditions, and suggest that economy of contraction should be calculated only from muscles undergoing isometric contractions.

## Data Availability Statement

The datasets generated for this study are available on request to the corresponding author.

## Ethics Statement

The animal study was reviewed and approved by The University of Auckland Animal Ethics Committee under the Approval Number R2006.

## Author Contributions

J-CH performed the experiments and analyzed the data. All authors contributed to the conception and design of the study, interpretation and discussion of the data, drafting of the manuscript, and approved the final version of the manuscript.

## Conflict of Interest

The authors declare that the research was conducted in the absence of any commercial or financial relationships that could be construed as a potential conflict of interest.
